# Apolipoprotein E Fragmentation within Lewy Bodies of the Human Parkinson’s Disease Brain

**DOI:** 10.23937/IJND-2017/1710002

**Published:** 2018-02-23

**Authors:** Troy T Rohn, Jacob M Mack

**Affiliations:** Department of Biological Sciences, Boise State University, USA

**Keywords:** Apolipoprotein E, Protease, Parkinson’s disease, Lewy bodies, Oligodendrocytes, Nuclear localization

## Abstract

Although harboring the Apolipoprotein E4 (APOE4) allele is a well-known risk factor in Alzheimer’s disease (AD), whether a similar risk holds true for Parkinson’s disease (PD) is currently not known. To investigate whether apoE pathology is present in PD, an immunohistochemical study was undertaken with fixed, human PD brain sections from the substantia nigra utilizing a recently characterized antibody that detects an amino-terminal fragment of apoE. This antibody, termed the apoE cleavage fragment p17 (nApoECFp17) antibody specifically detects an amino-terminal 17 kDa fragment of apoE without reacting with full-length forms of the protein. Application of this antibody revealed the presence of this fragment in Lewy bodies in all cases examined. Colocalization of nApoECFp17 with an antibody to alpha-synuclein (α-Syn), which served as a general marker for Lewy bodies, indicated the presence of this apoE fragment in 87.5% of all identified Lewy bodies. In addition, localization of nApoECFp17 was also evident within oligodendrocytes, the nucleus of melatonin-containing neurons, and blood vessels. Conversely, little staining was observed in the substantia nigra from Pick’s disease or in the frontal cortex of dementia with Lewy bodies (DLB) cases, suggesting a specificity for nApoECFp17 immunoreactivity in PD. Collectively, these data have identified widespread evidence for apoE fragmentation in the human PD brain and documented for the first time the presence of apoE within Lewy bodies, the major pathological marker for this neurodegenerative disease.

## Introduction

Parkinson’s disease (PD) is the second most common age-related, progressive neurodegenerative disorder after Alzheimer’s disease (AD) and is clinically characterized as a movement disorder presenting with rigidity, resting tremor, disturbances in balance and slowness in movement [1]. Pathologically, PD is characterized by the presence of intraneuronal inclusions termed Lewy bodies. Lewy bodies are circular, intracytoplasmic inclusions that contain abnormally truncated proteins, including alpha-synuclein (α-Syn) [2,3].

Human apolipoprotein E (apoE) is a polymorphic gene that includes apoE2, apoE3, and apoE4, which differ by single amino acid substitutions involving cysteine-arginine replacements at positions 112 and 158 [4]. Although inheritance of *APOE4* allele is a well-known risk factor for dementia, whether it poses a similar risk in PD has yielded conflicting results (for examples see [5–12]).

We recently synthesized a site-directed cleavage antibody that specifically recognizes an amino-terminal fragment of 17 kDa (p17) following cleavage after D151 of the mature, full-length form of apoE [13]. This antibody, which we termed the amino-terminal apoE cleavage fragment antibody (nApoECFp17 antibody) is highly specific for this fragment and shows no immunoreactivity to the full-length, 34 kDa form of the protein [13]. *In situ*, we demonstrated widespread labeling of this antibody in the AD brain and most surprising, strong localization within glia cells [13]. We further showed that a recombinantly produced fragment of apoE4, 1–151 was taken up by the microglia cell line, BV2, following extracellular treatment and trafficked to the nucleus causing significant toxicity.

The purpose of the current study was to determine whether the presence of this fragment could also be identified in the human PD brain. Our results showed widespread immunoreactivity of nApoECFp17 in all cases examined and, for the first time, documented the presence of this amino-terminal fragment within Lewy bodies and oligodendrocytes in the substantia nigra. The presence of apoE fragmentation in postmortem PD brain sections supports the hypothesis that apoE fragmentation may be a common event, as has been previously shown in AD.

## Methods

### Materials

The nApoECFp17 in house, rabbit, affinity-purified polyclonal antibody has been previously characterized and is specific for the 17 kDa amino-terminal fragment of apoE following cleavage after amino acid residue D151 [13]. This antibody does not react with full-length forms of apoE and reacts with the p17 fragment following cleavage from both full-length apoE3 and E4 [13]. The monoclonal antibody, Olig-1, was purchased from EMD Millipore (Billerica, MA). The monoclonal anti-alpha- synuclein antibody (LB 509) was purchased from Abcam (Cambridge, MA). All antibodies were used at a 1:100 dilution.

### Immunohistochemistry

Autopsy brain tissue from five neuropathologically confirmed PD cases, two dementia with Lewy body (DLB) cases, and three Pick’s disease cases were studied. Case demographics are presented in Table 1. Fixed substantia nigra or frontal cortex tissue sections used in this study were provided by the Institute for Memory Impairments and Neurological Disorders at the University of California, Irvine. Free-floating 40 µm-thick sections were used for immunohistochemical studies as previously described [14]. The tissue was provided without identifiers and thus is not considered human subject research by NIH criteria.

For single labeling, all sections were washed with 0.1 M Tris-buffered saline (TBS), pH 7.4, and then pretreated with 3% hydrogen peroxide in 10% methanol to block endogenous peroxidase activity. Sections were subsequently washed in TBS with 0.1% Triton X-100 (TBS-A) and then blocked for thirty minutes in TBS-A with 3% bovine serum albumin (TBS-B). Sections were further incubated overnight at room temperature with the nApoECF antibody (1:100). Following two washes with TBS-A and a wash in TBS-B, sections were incubated in anti-rabbit or mouse biotinylated anti-IgG (1 hour) and then in avidin biotin complex (1 hour) (ABC, Elite Immunoperoxidase, Vector Laboratories, Burlingame, CA, USA). The primary antibody was visualized using Blue SG substrate (Vector Laboratories). To ensure antibody binding was specific, controls were performed that included either absence of the primary or secondary antibodies during the staining procedure. No staining was observed under these conditions.

### Immunofluorescence microscopy

Immunofluorescence studies were performed by incubating sections with primary antibodies overnight at a room temperature, followed by secondary anti-rabbit or mouse biotinylated anti-IgG (1 hour) and then in ABC (1 hour). Primary antibodies utilized included the nApoECFp17 (1:100), Olig-1 (mouse monoclonal, 1:100) and α-Syn (mouse monoclonal, 1:100). For immunofluorescence co-localization studies, antigen visualization was accomplished using an Alexa fluor 488-labeled tyramide (green, Ex/Em = 495/519) for one label and streptavidin Alexa fluor 555 (red, Ex/Em = 555/565) for the second label, both from Invitrogen (Carlsbad, CA). For microscopic observation and photomicrography of the Blue SG-labeled and fluorescent sections, an Olympus BX60 microscope with fluorescence capability equipped with a Magnafire SP software system for photomicrography was employed. The fluorescent molecules were excited with a 100-W mercury lamp. Fluorescent-labeled molecules were detected using a filter set having a 460–500-nm wavelength band pass excitation filter, a 505-nm dichroic beam splitter, and a 510–560-nm band pass emission filter. To ensure that observed antibody staining was specific and not due to autofluorescence, all sections were carefully examined by fluorescence before and after labeling. The presence of Lewy bodies was only apparent following specific labeling by either the nApoECFp17 or α-Syn antibodies.

### Confocal microscopy

For confocal immunofluorescence imaging, the primary antibodies were visualized with secondary antibodies tagged with either Alexa Fluor 488 or Alexa Fluor 555 (Invitrogen, Carlsbad, CA.) Images were taken with the Zeiss LSM 510 Metasystem combined with the Zeiss Axiovert Observer Z1 inverted microscope and ZEN 2009 imaging software (Carl Zeiss, Inc., Thornwood, NY). Confocal Z-stack and single plane images were acquired with an Argon (488 nm) and a HeNe (543 nm) laser source. Z-stacks images were acquired using a 20× Plan-Apochromat (NA 0.8) objective, emission band passes of 505–550 nm for the detection of the nApoECFp17 antibody (green channel, Alexa Fluor 488) and 550–600 nm for both the detection of Olig-1 (red channel, Alexa Fluor 555) and α-Syn (red, Alexa Fluor 555). All images displayed are 2-D maximal intensity projections generated acquired Z-stacks. Single plane images were acquired with a 63× Plan-Apochromat oil-immersion objective (NA 1.4) with emission long pass of 505 nm for the detection of the nApoECFp17 antibody (green channel, Alexa Fluor 488) and 550–600 nm for the detection of either Olig-1 or α-Syn (red channel, Alexa Fluor 555).

### Statistical analysis

To determine the percent colocalization, a quantitative analysis was performed as described previously [13] by taking 40× immunofluorescence, overlapping images from three different fields in the substantia nigra in three separate PD cases. Capturing was accomplished by using a 2.5× photo eyepiece, and a Sony high resolution CCD video camera (XC-77). As an example, to determine the percent co-localization between nApoECFp17 and Olig-1, photographs were analyzed by counting the number of Olig-1-positive oligodendrocytes alone per 40× field for each case, and the number of cells labeled with both Olig-1 and nApoECFp17. Data are representative of the average number (± S.E.M.) of each antibody alone or co-localized with both antibodies in each 40× field (3 fields total for 3 different cases). Statistical differences in this study were determined using Student’s two-tailed T-test employing Microsoft Office Excel.

## Results

Previous characterization of the nApoECFp17 antibody indicated that it is highly specific for a 17 kDa amino-terminal fragment of apoE [13]. This in-house antibody recognizes the N-terminal upstream neoepitope fragment of apoE3 and E4 that would be generated following cleavage after the terminal aspartic acid residue at position D151 of the full-length protein. Importantly, the antibody shows no reactivity to full-length forms of apoE [13]. To determine if amino-terminal fragments of apoE can be detected in the human PD brain, an immunohistochemical study utilizing the nApoECFp17 antibody was initiated utilizing fixed substantia nigra brain sections from confirmed PD cases. Case demographics used in this study are presented in Table 1.

**Figure 1:**
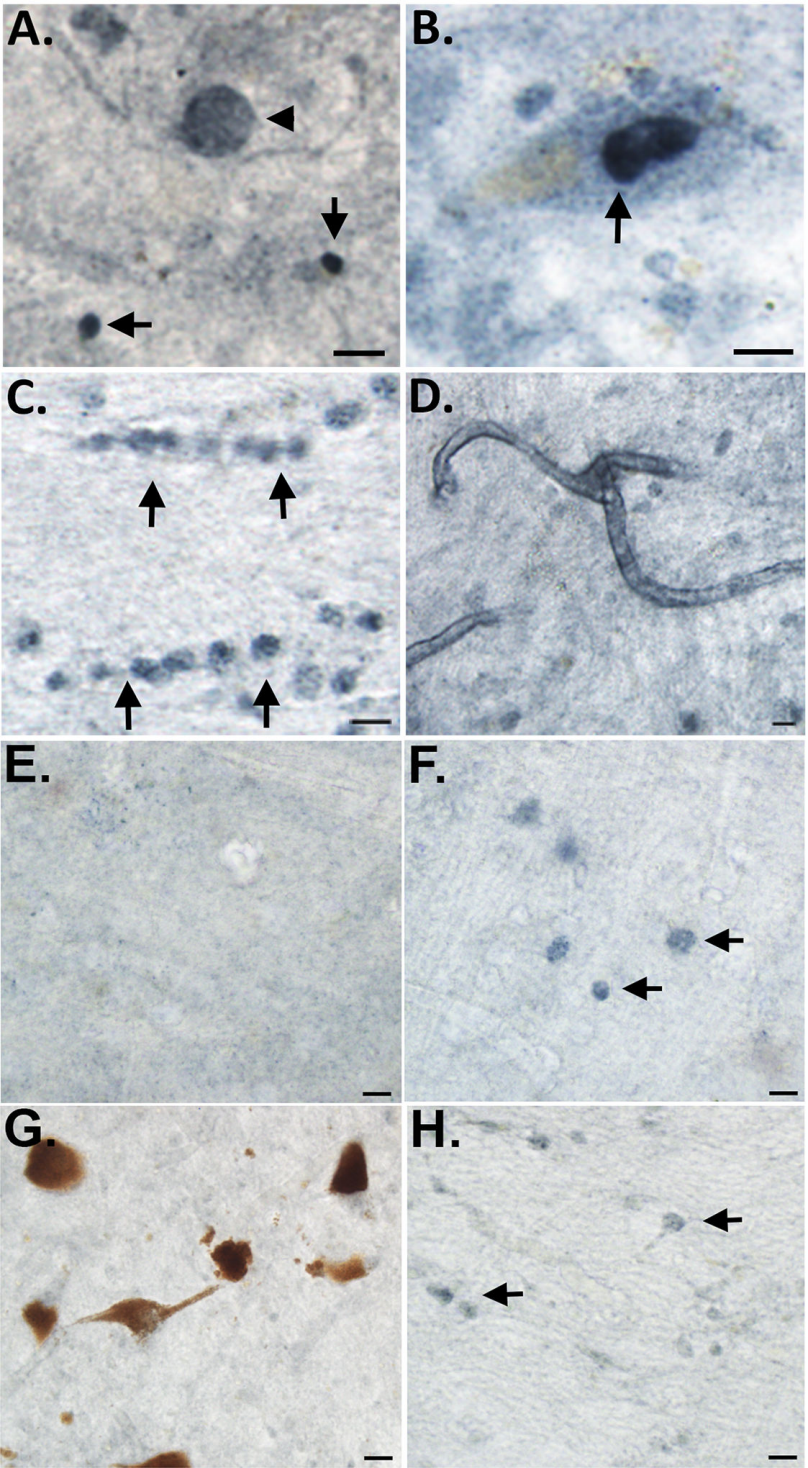
Detection of fragmented apoE in the substantia nigra of the PD brain Representation bright-field staining in PD substantia nigra tissue sections following application of the nApoeCFp17 antibody (1:100) **(A)** Representative immunostaining of an apparent Lewy body (arrowhead) by nApoECFp17 as well as smaller circular structures (arrows); **(B–D):** Staining was also observed within the nucleus of melanin-containing neurons (B, arrow), in oligodendrocytes in white matter (arrows, C), and along blood vessels (D); **(E and F):** Representative immunostaining utilizing the nApoECFp17 (1:100) antibody in frontal cortex sections from two DLB cases indicating a relative lack of labeling except weakly within apparent oligodendrocytes in white matter (F, arrows); **(G and H):** Representative staining with the nApoECFp17 antibody (1:100) within the substantia nigra of two separate Pick’s disease cases indicating a lack of immunoreactivity. All scale bars represent 10 µm.

As an initial step, we screened all five cases for nApoECFp17 immunoreactivity using bright-field microscopy. Following application of the nApoECFp17 antibody, widespread labeling was found in all cases examined with prominent labeling observed in apparent Lewy bodies, nuclei of melanin-containing neurons, oligodendrocytes, and along blood vessels (Figure 1). Putative staining within oligodendrocytes is based on the fact this staining was predominantly localized within white matter of substantia nigra sections and cells that were labeled displayed a linear pattern of appearance that is typical of oligodendrocytes. With the exception of Lewy body labeling, these findings are similar to what was previously documented using the nApoECFp17 antibody in the AD brain [13]. To determine whether the observed labeling of nApoECFp17 was specific to PD, experiments were undertaken utilizing frontal cortex sections from two DLB cases. As shown in Figure 1E and Figure 1F, we were unable to identify labeling within apparent Lewy bodies although weak labeling within glial cells was apparent (Figure 1E and Figure 1F, arrows). In addition, as a negative control, we also screened the substantia nigra from three Pick’s disease cases. Unlike PD and DLB which are classified as pure α-synucleinopathies, Pick’s disease presents with tauopathy associated with frontotemporal lobe atrophy [15]. In all three Pick’s cases, we observed a paucity of nApoECFp17 labeling with only weak glial staining in white matter (Figure 1G and Figure 1H, arrows). Taken together, these data support the specificity of nApoECFp17 pathology in the substantia nigra of the human PD brain.

**Figure 2:**
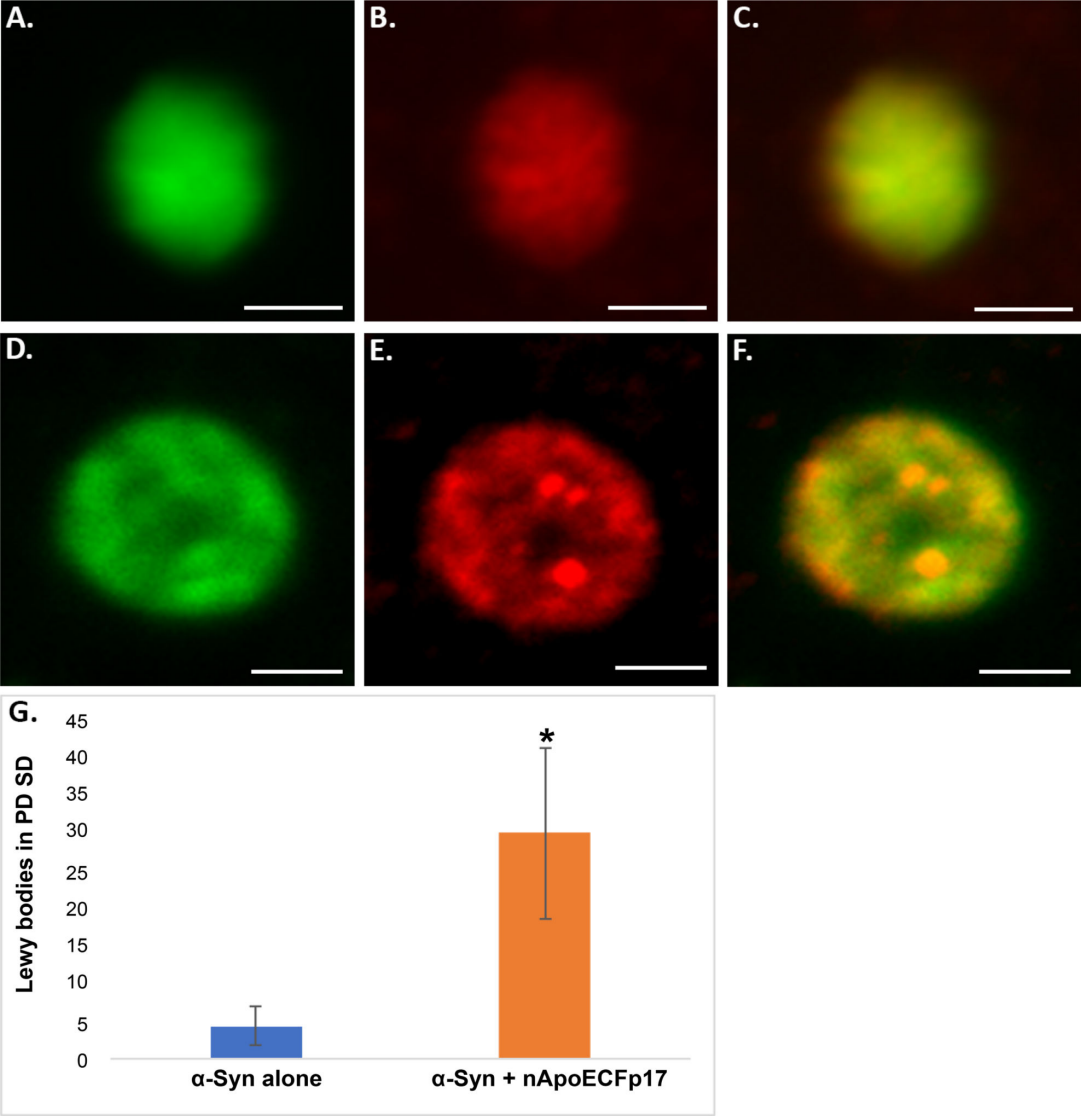
Localization of an amino-terminal fragment of apoE within Lewy bodies of the human PD brain (A–F) Representation images from confocal immunofluorescence in two different PD cases utilizing antibodies nApoECFp17 (A and D), α-Syn (B and E), with the merged images shown in (C and F). Strong co-localization of the two antibodies was observed in Lewy bodies of the PD brain; **(G):** Quantification of the number of Lewy bodies doubled-labeled with nApoECFp17 and α-Syn indicated co-localization in 87.5% of the total number of Lewy bodies identified in the substantia nigra. Data depict the number of α-Syn-labeled Lewy bodies alone (blue bar) and the number of Lewy bodies labeled with both α-Syn and nApoECFp17 (orange bar) identified in substantia nigra PD sections by immunofluorescence microscopy (n = 3 different PD cases ± S.D.). All scale bars represent 5 µm. Asterisk denotes significant difference, p = 0.018.

To confirm the presence of nApoECFp17 immunoreactivity within Lewy bodies, double-label confocal immunofluorescence studies were undertaken using α-Syn, a standard marker for Lewy bodies. As depicted in Figure 2A, Figure 2B, Figure 2C, Figure 2D, Figure 2E and Figure 2F, Lewy bodies showed strong co-localization of these two antibodies and quantification indicated that 87.5% of identified Lewy bodies also were immunopositive for nApoECFp17 (Figure 2G).

**Figure 3:**
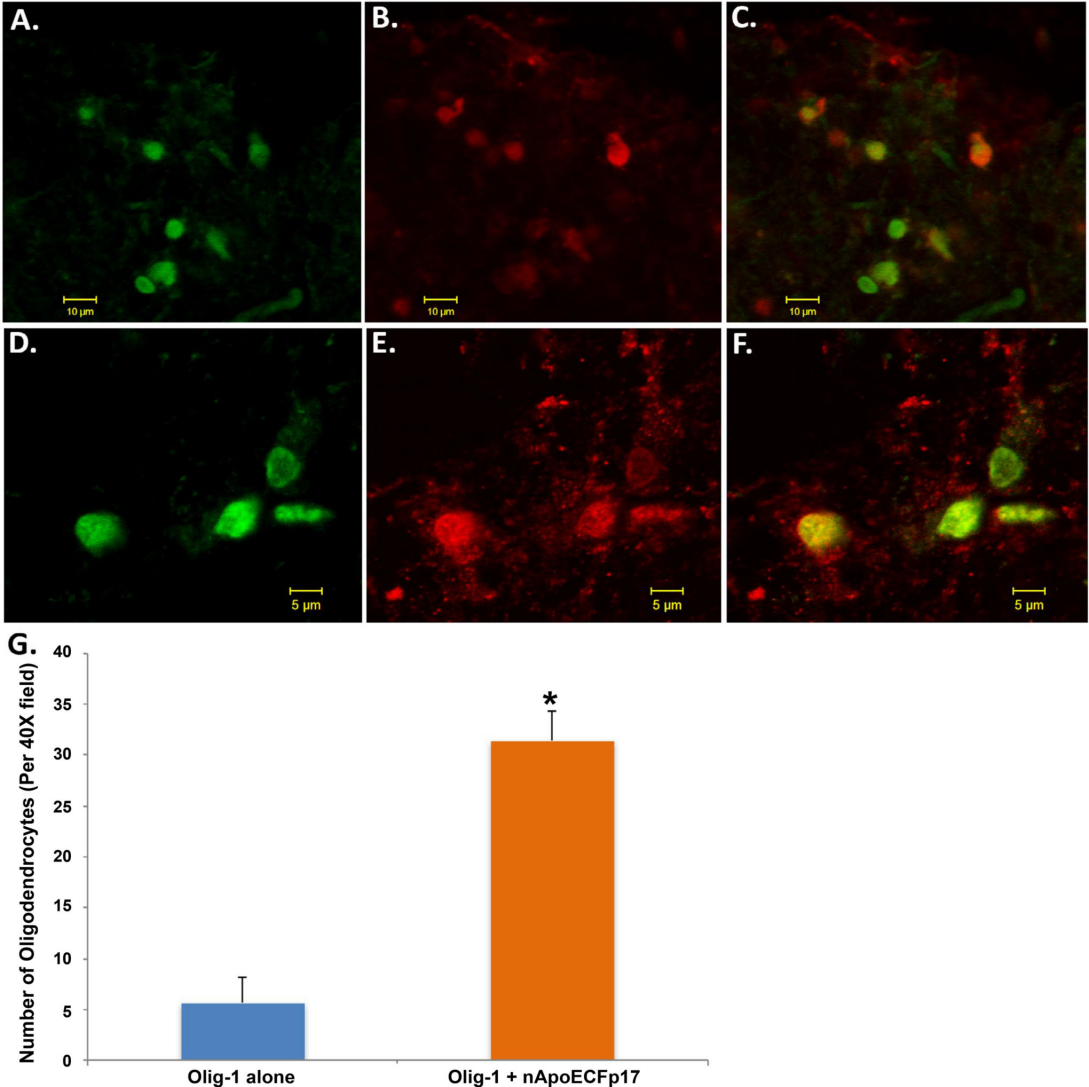
Localization of an amino-terminal fragment of apoE within oligodendrocytes of the human PD brain (A–F) Representation images from confocal immunofluorescence in two different PD cases utilizing antibodies nApoECFp17 (A and D), Olig-1 (B and E), with the merged images shown in (C and F). Strong co-localization of the two antibodies was observed in oligodendrocytes of the PD brain; **(G):** Quantification of the number of oligodendrocytes doubled-labeled with nApoECFp17 and Olig-1 indicated co-localization in 84.7% of the total number of oligodendrocytes identified in respective fields. Data show the number of Olig-1-labeled-oligodendrocytes alone (blue bar) and the number of oligodendrocytes-labeled with both Olig-1 and nApoECFp17 (orange bar) identified in a 40× field within substantia nigra PD sections by immunofluorescence microscopy (n = 3 fields for 3 different PD cases ± S.E.M.). Asterisk denotes significant difference, p = 1.06 × 10^−8^.

In a previous study, a major finding was the presence of nApoECFp17 within the nuclei of glia cells of the AD brain [13]. Preliminary observations (Figure 1) suggested that a similar finding also occurs in the PD brain including possible labeling with oligodendrocytes. To confirm labeling within oligodendrocytes, double-labeling immunofluorescence confocal studies were carried out using the standard marker, Olig-1. Co-localization between Olig-1 and the nApoECFp17 antibody was observed in white matter of substantia nigra sections (Figure 3). Interestingly, while the nApoECFp17 antibody labeling was primarily nuclear, Olig-1 labeling was more widespread and appeared to label processes that were fragmented and damaged (Figure 3E and Figure 3F). Quantification indicated that of the total number of oligodendrocytes identified by Olig-1, 84.7% also were labeled with the nApoECFp17 antibody (Figure 3G).

## Discussion

The purpose of this study was to examine whether apoE pathology could be documented in the human PD brain. One of the major findings of the current study was the identification of this amino-terminal fragment of apoE within Lewy bodies of the PD brain. In addition, the lack of labeling of the nApoECFp17 antibody in frontal cortex of DLB cases and in the substantia nigra of Pick’s disease cases suggests a specificity of nApoECFp17 pathology in PD. To our knowledge, this is the first time apoE pathology has been identified within Lewy bodies, which have been previously described as failed proteasomes containing aggregated α-Syn and ubiquitinated proteins [2,16]. The presence of truncated apoE within Lewy bodies is significant as these intraneuronal structures are the major pathological markers of PD and are associated with cognitive impairment in PD and DLB [17,18].

Based on the presence of truncated and aggregated proteins, including TDP-43 [19], α-Syn, and tau [2,20], it is plausible that Lewy bodies may serve a protective function by sequestering potentially cytotoxic proteins. In the context of the current findings, previous studies have shown that amino-terminal fragments of apoE4 are cytotoxic and promote neurodegeneration [13,21,22]. Thus, apoE4 has been postulated to enhance dementia risk at the molecular level through a possible toxic-gain of function. In this manner, previous studies have shown that apoE4 is much more susceptible to proteolysis than apoE3 or E2 and the amino-terminal fragments that are generated are in fact neurotoxic when exposed to cultured cells or expressed in transgenic mouse models [21– 23]. In our recent study, we demonstrated that exposure of BV2 microglial cells to an amino-terminal fragment of apoE4 (1–151) lead to significant neurotoxicity and cell death [13]. We hypothesize that if a similar process is occurring in the PD brain as documented by the presence of nApoECFp17, this could promote the underlying neurodegeneration associated with this disease. In addition, cognitive impairment is prevalent in PD, affecting 15–20% of patients [24], and recent studies have supported *APOE4* allele carrier frequency was significantly higher in PD with executive dysfunction [25,26]. Therefore, apoE4 may also contribute to cognitive impairment associated with PD as previously suggested [27,28]. Our current results demonstrating the presence of fragmented apoE adds to the growing body of studies implicating this protein in the pathogenesis of PD.

The accumulation of aggregated forms of α-Syn protein into Lewy bodies is one of the characteristic features of PD [2]. A question raised from the current findings is: What is the potential significance between α-Syn and nApoECF (1–151) detected by our nApoECFp17 antibody? In a study by Emamzadeh, et al., the authors examined the effects of different isoforms of apoE on aggregation of α-Syn and found that aggregation is stimulated by all isoforms, with apoE4 showing the greatest stimulatory effect [29]. Based on our documentation of apoE pathology in the PD brain, together these studies suggest that low levels of apoE may seed α-Syn aggregation, which could potentially lead to a pathway of α-Syn-induced neurodegeneration.

An additional finding of this study was the presence of truncated apoE within oligodendrocytes in white matter of the PD brain. Interesting, many of these labeled oligodendrocytes appeared to display fragmented processes (Figure 3E and Figure 3F), an indication that these cells may be undergoing degeneration. Because of the critical role oligodendrocytes play in myelination of axons in the CNS, the degeneration of oligodendrocytes may contribute to the extrapyramidal symptoms associated with PD.

It is important to point out a couple of caveats of the current study. First, we were unable to obtain information on specific *APOE4* allelic genotype for the PD cases used in this study. Therefore, we were unable to dissect out any possible influence of harboring the *APOE4* allele on our findings. Second, the primary antibody used in this study, nApoECFp17, specifically immunolabels a 17 kDa amino-terminal fragment of apoE without reacting to full-length forms of the protein [13]. Because apoE2, E3, and E4 differ by single amino acid substitutions involving cysteine-arginine replacements at positions 112 and 158 [4], the nApoECFp17 does not discriminate between the different isoforms and will readily recognize the p17 fragment derived from full-length apoE3 and E4 [13].

## Conclusion

The presence of truncated apoE as shown in the present study supports the notion that these species are being sequestered within Lewy bodies. However, whether truncated apoE in PD is a cause or effect of the disease will require further studies. Ideally, a comprehensive study that would include examining specific *APOE4* allelic cases would need to be undertaken to examine whether fragmentation happens to a greater degree in *APOE4* carriers.

## Figures and Tables

**Table 1:** Case demographics for immunohistochemistry.

Case	Sex	Age	PMI (hrs)	NPD × 1	Cause of Death
1	M	64	2.25	PD	Respiratory Failure
2	M	71	3.5	PD	Cardiopulmonary Arrest
3	F	88	3	PD	Pneumonia
4	F	87	2.3	PD	Unknown
5	M	69	4.5	PD	Cardiopulmonary Arrest
6	M	72	5.5	DLB	Respiratory Failure
7	M	83	9.5	DLB	Cardiopulmonary Arrest
8	M	66	5	Pick’s	End-stage Pick’s disease
9	F	68	3.15	Pick’s	Acute Pulmonary Embolism
10	F	76	3.3	Pick’s	Cardiorespiratory Arrest

PMI = Postmortem Interval; NPD × 1 = Primary pathological diagnosis.

## Abbreviations

ApoE: Apolipoprotein E
nApoECFp17: ApoE Amino-terminal Cleavage Fragment p17
PD: Parkinson’s Disease
AD: Alzheimer’s Disease
α-Syn: Alpha-synuclein
TBS: Tris-buffered Saline
DLB: Dementia with Lewy Bodies
